# Genomic profiling in ovarian cancer retreated with platinum based chemotherapy presented homologous recombination deficiency and copy number imbalances of *CCNE1* and *RB1* genes

**DOI:** 10.1186/s12885-019-5622-4

**Published:** 2019-05-06

**Authors:** Alexandre A. B. A. da Costa, Luisa M. do Canto, Simon Jonas Larsen, Adriana Regina Gonçalves Ribeiro, Carlos Eduardo Stecca, Annabeth Høgh Petersen, Mads M. Aagaard, Louise de Brot, Jan Baumbach, Glauco Baiocchi, Maria Isabel Achatz, Silvia Regina Rogatto

**Affiliations:** 1 Department of Medical Oncology, AC Camargo Cancer Center, Rua Professor Antonio Prudente 211, São Paulo, CEP: 01509-010 Brazil; 2 CIPE - AC Camargo Cancer Center, São Paulo, Brazil; 3 Dept of Clinical Genetics, Vejle Hospital, Institute of Regional Health Research, University of Southern Denmark, Vejle, DK Denmark; 4 Dept of Mathematics and Computer Science, University of Southern Denmark, Odense, DK Denmark; 5 Dept of Pathology, AC Camargo Cancer Center, São Paulo, Brazil; 6 Chair of Experimental Bioinformatics, TUM School of Life Sciences Weihenstephan Technical University of Munich, Munich, Germany; 7 Dept of Gynecologic Oncology, AC Camargo Cancer Center, São Paulo, Brazil; 8 Centro de Oncologia, Hospital Sírio-Libanês, São Paulo, Brazil

**Keywords:** Ovarian cancer, Homologous recombination deficiency, Target-next generation sequencing, Copy number alterations, Treatment response, *CCNE1* gains, *RB1* loss

## Abstract

**Background:**

Ovarian carcinomas presenting homologous recombination deficiency (HRD), which is observed in about 50% of cases, are more sensitive to platinum and PARP inhibitor therapies. Although platinum resistant disease has a low chance to be responsive to platinum-based chemotherapy, a set of patients is retreated with platinum and some of them are responsive. In this study, we evaluated copy number alterations, HR gene mutations and HR deficiency scores in ovarian cancer patients with prolonged platinum sensitivity.

**Methods:**

In this retrospective study (2005 to 2014), we selected 31 patients with platinum resistant ovarian cancer retreated with platinum therapy. Copy number alterations and HR scores were evaluated using the OncoScan® FFPE platform in 15 cases. The mutational profile of 24 genes was investigated by targeted-NGS.

**Results:**

The median values of the four HRD scores were higher in responders (LOH = 15, LST = 28, tAI = 33, CS = 84) compared with non-responders (LOH = 7.5, LST = 17.5, tAI = 23, CS = 47). Patients with high LOH, LST, tAI and CS scores had better response rates, although these differences were not statistically significant. Response rate to platinum retreatment was 22% in patients with *CCNE1* gains and 83.5% in patients with no *CCNE1* gains (*p* = 0.041). Furthermore, response rate was 54.5% in patients with *RB1* loss and 25% in patients without *RB1* loss (*p* = 0.569). Patients with *CCNE1* gains showed a worse progression free survival (PFS = 11.1 months vs 3.7 months; *p* = 0.008) and a shorter overall survival (OS = 39.3 months vs 7.1 months; *p* = 0.007) in comparison with patients with no *CCNE1* gains. Patients with *RB1* loss had better PFS (9.0 months vs 2.6 months; *p* = 0.093) and OS (27.4 months vs 3.6 months; *p* = 0.025) compared with cases with no *RB1* loss. Four tumor samples were *BRCA* mutated and tumor mutations were not associated with response to treatment.

**Conclusions:**

HR deficiency was found in 60% of our cases and HRD medium values were higher in responders than in non-responders. Despite the small number of patients tested, *CCNE1* gain and *RB1* loss discriminate patients with tumors extremely sensitive to platinum retreatment.

**Electronic supplementary material:**

The online version of this article (10.1186/s12885-019-5622-4) contains supplementary material, which is available to authorized users.

## Background

Ovarian cancer, the most lethal gynecologic cancer, is expected to account for 14,700 deaths in the USA in 2018 [1]. High grade serous carcinoma (HGSC), the most frequent histological type [2], is molecularly characterized by a few recurrent mutations, including in *TP53* (almost all tumors) and *BRCA1* or *BRC2* genes. HGSCs present high genomic instability with copy number alterations (CNA) affecting a large fraction of the genome [3]. Approximately 50% of these tumors are characterized by homologous recombination (HR) deficiency, which has been associated with *BRCA1* or *BRCA2* germline or somatic mutations (20 and 5% of cases, respectively), *BRCA1* promoter methylation (10% of cases), additional mutations in HR repair pathway genes and CNA in their regulators (*PTEN* and *EMSY*) [3].

Patients carrying tumors with *BRCA* mutation tend to have elevated sensitivity to platinum-based chemotherapy [4] and PARP inhibitors [5–8]. These cases have shown a better medium term prognosis [9] even if the cure rate and long term prognosis is unaltered [10]. Secondary mutations have been associated with resistance to PARP inhibitors [11, 12].

Two strategies have been used to identify tumors with HR deficiency or alterations in genes involved in the DNA repair system other than *BRCA1* and *BRCA2* mutations. The first approach is to identify mutations in genes related to HR pathway [13], based on next generation sequencing (NGS), which has become feasible with the fast development of sequencing technologies. The second is the identification of “genomic scars”, which are supposed to be a functional consequence of HR deficiency independently of its cause [14–17]. Clinical trials revealed that even patients with no HR deficiency, evaluated by two different HR deficiency scores, can achieve response to PARP inhibitors [8, 13]. These outcomes could be explained by other mechanisms of action than synthetic lethality of the HR pathway deficiency or failure in identifying the HR defect, or both.

In addition to the genomic scars of HR deficiency, gains or losses involving specific genes have also been associated with response to therapy in ovarian cancer [18]. Cyclin E1 and RB1 are cell-cycle proteins associated with the G1-S phase cell-cycle transition. *CCNE1* copy number gain is described in about 20% of HGSC and is seemingly rare in *BRCA* mutated tumors [3]. Tumors with *CCNE1* copy number gains are more resistant to platinum therapy [19] while *RB1* loss are associated with high sensitivity to platinum therapy [20, 21].

Platinum resistant disease has a low chance to be responsive to platinum-based chemotherapy. The standard treatment is monotherapy using different drugs than platinum salts [2]. In daily clinical practice and despite of the resistant profile, a set of patients is retreated with platinum therapy and some of them are responsive to platinum retreatment [22].

In this study, we sought to evaluate the association of HR pathway mutations, HR deficiency scores and *CCNE1* and *RB1* CNA with response to platinum retreatment in ovarian cancer patients in the platinum-resistant setting.

## Methods

### Patients

From 2005 to 2014, 405 patients with ovarian carcinoma were treated at AC Camargo Cancer Center, São Paulo, Brazil. Thirty-five of them presented platinum resistant recurrence and were retreated with platinum therapy. Patients with unavailable data regarding the platinum retreatment were excluded (4 patients) and a retrospective review of the medical records was performed (Additional file 1). Based on the quantity and quality of tumor DNA, 15 of 31 cases were selected for SNP array (OncoScan® FFPE, Thermo Fisher Scientific, Waltham, MA, USA) analyses, and 11 of them were also evaluated by targeted-next generation sequencing. Nine patients had the primary tumor naive of treatment, five patients had the tumor sample collected at platinum resistant recurrence, and one had the tumor sample collected at platinum sensitive recurrence. The study was conducted in accordance with the Declaration of Helsinki ethical guidelines and approved by the institutional Ethics Committee (CEP# 1933/14).

### Clinical data

Clinical features were retrieved from the medical records including age at diagnosis of platinum resistant recurrence, tumor histological subtype, family or personal history of ovarian and breast cancer, number of previous treatment lines, platinum free interval (PFI), and type of chemotherapy associated to platinum (Table 1).

**Table 1:** Clinical features of 31 patients with ovarian cancer who had previous platinum resistant relapse and were retreated with platinum

Clinical Characteristics	Number of cases (%)
Age (years old)	
< 65	21 (67.7)
> 65	10 (32.3)
Histology	
High grade serous carcinoma	21 (67.7)
Endometrioid	1 (3.2)
Clear cell carcinoma	1 (3.2)
Undifferentiated carcinoma	3 (9.7)
Carcinosarcoma	1 (3.2)
Mixed	1 (3.2)
Family history of ovarian or breast cancer	
No	19 (61.3)
Yes	9 (29.0)
Number of previous treatment lines	
2	10 (32.3)
3	7 (22.6)
4	4 (12.9)
5	6 (19.4)
6	3 (9.7)
8	1 (3.2)
Platinum free interval (months)	
< 12	21 (67.7)
> 12	10 (32.3)
Primary platinum resistance*	
No	12 (38.7)
Yes	19 (61.3)
Chemotherapy with platinum rechallenge	
Platinum + taxane	15 (48.4)
Platinum + gemcitabina	13 (41.9)
Platinum + doxorubicin	1 (3.2)
Platinum + ifosfamide	1 (3.2)
Monotherapy	1 (3.2)

*Primary platinum resistant disease was defined as a first recurrence with a platinum free interval less than 6 months. Patients without primary platinum resistant recurrence, presented platinum resistant disease at second or later recurrences

Recurrence was defined according to the GCIG (Gynecological Cancer Intergroup) criteria after the analysis of RECIST (Response Evaluation Criteria in Solid Tumors) [23, 24]. The CA125 levels were extracted from the medical records. The date of the earliest event was considered for progression. The recurrence detected within 6 months after the last platinum infusion was defined as platinum resistant recurrence. All recurrences that followed this first platinum resistant recurrence were also considered platinum resistant. Progression-free survival (PFS) was defined as the interval between the date of the beginning of the platinum retreatment and disease progression or death by any cause. Overall survival (OS) was defined as the interval between the dates of the beginning of the platinum retreatment and death by any cause. The interval between the date of the last platinum compound infusion and the disease progression that preceded platinum retreatment was used to define the platinum-free interval (PFI).

The CA125 expression levels and the response to platinum retreatment data were retrieved from the medical records. The image reports were also collected. GCIG criteria were used to evaluate RECIST and CA125 data [23, 24]. In accordance, each case was categorized as having “response” (complete or partial response) or “no response” (stable disease or disease progression).

### DNA extraction

Ten μm paraffin embedded tissue sections were deparaffinized with xylene, washed with descending concentrations of ethanol and water ultra-pure sterile. DNA was extracted using QIAamp DNA FFPE Tissue Kit (Qiagen, Valencia, CA) according to the manufacturer’s instructions. DNA integrity was evaluated using Agilent Genomic DNA ScreenTape (Agilent Techologies, Santa Clara, USA) and quantified using a Qubit Fluorometer (Life Technologies, Carlsbad, CA).

### OncoScan assay

The OncoScan® FFPE platform (Thermo Fisher Scientific) allows the identification of over 200,000 SNPs and the detection of 74 somatic mutations in nine genes (*BRAF, KRAS, EGFR, IDH1, IDH2, PTEN, PIK3CA, NRAS and TP53*). However, we focused only in CNAs. The SNP array assay was performed according to the recommended protocol using 80 ng of genomic DNA. After the hybridization (18 h), the arrays were stained and washed (GeneChip® Fluidics Station 450) and loaded into the GeneChip® Scanner 3000 7G (Thermo Fisher Scientific/Affymetrix). The CEL files were generated by Affymetrix Gene Chip Comand Console® software (v. 4.0) and processed by OncoScan Console software (v. 1.3) resulting in OSCHP files and QC metrics.

Data generated from the SNP array was used to calculate previously defined scores of homologous recombination deficiency: loss of heterozygosity (LOH) [16], telomeric allelic imbalance (tAI) [14], large scale transition (LST) [15], and Composite Score (CS) (the sum of LOH, tAI and LST scores) [25]. LOH was calculated as the number of LOH regions spanning at least 15 MB but not involving the entire chromosome. The number of regions with allelic imbalance extending to one of the telomeres but not crossing the centromere, after filtering regions shorter than 11 MB or spanning less than 500 probes, was defined as tAI. The LST score was defined as the number of breakpoints between regions spanning at least 10 MB within a distance of maximum 3 MB. HRD was computed as LOH + tAI + LSTm (adjusted LST score). According to Timms et al. [26], the HRD score introduced by Telli et al. [25] increases with ploidy in both intact and deficient samples. The adjusted LST score is calculated as [LSTm = LST-15.5P], in which P is the tumor ploidy. Using logistic regression analysis, the constant 15.5 was derived to provide the best separation between intact and deficient samples. ASCAT [27] was used for inferring tumor ploidy, calculating allele-specific copy numbers and segmentation. The cut-off values were previously established as HRD markers: > 10 for LOH [16], > 42 for CS [25] and > 15 for near-diploid tumors or > 20 for near-tetraploid tumors for LST scores [15]. The tAI median value was used as cut-off to consider it as HRD marker.

*CCNE1* and *RB1* CNAs were evaluated using the SNP array with a resolution of 1 probe per 50Kb for both genes. The average copy number for all probes covering each gene **>** 2.0 and < 2.0 was defined as gain or loss, respectively.

### Target enrichment next generation sequencing (tNGS)

The HaloPlexHS target enrichment technology (Illumina 100 custom design with a 229,506 bp region of interest) (Agilent Technologies, 2016) was used to investigate mutations in 24 genes (*BRCA1, BRCA2, TP53, BRIP1, CDH1, PALB2, RAD51C, RAD51D, XRCC2, MLH1, MSH2, MSH6, PMS2, EPCAM, APC, MUTYH, BMPR1A, SMAD4, STK11, PTEN, POLD1, POLE, NTHL1* and *VHL*). The fraction of bases in the region of interest covered 98.76% of the target region. HaloPlexHS libraries were constructed according to manufacturer’s protocol v.B0 (Agilent Technologies, June 2015). Indexes were incorporated for each sample during enrichment, allowing samples to be multiplexed before sequencing. A total of 11 HaloPlexHS libraries were validated on a TapeStation using High Sensitivity screentape (Agilent Technologies). After enrichment, HaloPlexHS libraries were diluted to 10 nM, pooled, denatured and subjected to paired-end (2× 150 bp), single index (8 bp) reversible terminator-based DNA sequencing on a NextSeq550 (Illumina) using a mid-output kit and loading 1.8 pM of denatured library pool.

For each sequenced sample, the raw FastQ files were trimmed with TrimGalore (v. 0.4.2), subsequently mapped to the GRCh37/hg19 human reference genome using MOSAIK (v. 2.2.26) and converted to BAM using Sambamba (v. 0.6.3). The BAM file for each sample was preprocessed with Genome Analysis Toolkit (GATK v. 3.6; local realignment around indels and base quality score recalibration), prior to variant calling. General alignment statistics (e.g. number of aligned reads etc.) was generated with BAMtools (v.2.3.0). Target specific alignment statistics (i.e. per base−/region−/gene−/sample-coverage and coverage percentage of ROIs), were obtained using GATK DepthOfCoverage. Variant calling was performed using the variantcallers: GATK HaplotypeCaller in genomic VCF mode, GATK UnifiedGenotyper (GATK v. 3.6), FreeBayes (v. 1.1.0) and finally PLATYPUS (v. 0.8.1). A single multisample VCF file comprising all analyzed samples was generated for each variantcaller. Four multisample VCF files were subsequently merged to produce a single multisample VCF file.

The variants were annotated with Ingenuity Variant Analysis (Qiagen), SnpEff (v 4.1c), ANNOVAR (v July 2017) and VariantTools (v.2.3), using build-in and custom annotation tracks. Additional file 2 summarizes the pipeline used to evaluate the generated variants.

### Statistical analysis

Statistical analyzes were performed using the SPSS (v. 21.0; SPSS, Chicago, IL, USA) software, adopting a two-tailed *P* < 0.05 value as significant. The association among response rate to platinum retreatment in patients with platinum resistant ovarian cancer and the HRD scores, all mutations, *CCNE1* CN gains and *RB1* CN losses, were investigated using Mann-Whitney and Fischer’s Exact tests. Correlation analysis between the HRD scores was tested using Pearson’s coefficient and linear regression. Overall survival and progression free survival analyses were performed using Kaplan-Meier and log-rank test.

## Results

A total of 31 patients with platinum resistant ovarian cancer was retreated with platinum therapy during a period of 10 years (2005 to 2014) at AC Camargo Cancer Center, SP, Brazil. High grade serous ovarian cancer was found in 68% of all cases, half of the patients received four or more prior treatment lines and 61.3% of the cases presented primary platinum resistant disease. All patients except three, were treated with taxane or gemcitabine in combination with platinum. Table 1 summarizes the clinical and pathological features of these 31 patients.

### Genomic alterations

Fifteen of 31 ovarian tumors samples were investigated for genomic alterations. Clinical and molecular data are summarized in Table 2. Twelve cases were HGSC, two undifferentiated carcinomas and one high grade endometroid ovarian carcinoma. The median HRD scores were: tAI = 31, LOH = 9.0, LST = 24 and CS = 64 (details in Table 3). Two undifferentiated carcinomas and the high grade endometrioid tumors showed high HRD scores. Seven cases presented high scores for tAI, seven for LOH, nine for LST and nine cases for CS. Seven of 15 cases presented high values for all scores. Overall, a strong to moderate correlation was observed among the HRD scores (Additional file 3).

**Table 2:** Clinical features, response to platinum retreatment and molecular findings of 15 ovarian cancer patients^a^

ID	Histology	Response	HRD	*TP53*	*BRCA1*	*BRCA2*	Other Mutations	Family History	*CCNE* CN	*RB1* CN
1	HGSC	CR	No	c.782 + 1G > A	WT	WT	WT	Yes	2.3	1.9
2	HGSC	PR	No	WT	WT	WT	WT	No	2.9	1.8
3	HGSC	SD	No	c.672 + 1G > T	WT	WT	*XRCC2* c.643C > T *APC* c136G > T	Yes	4.0	1.9
4	HGSC	SD	Yes	nd	nd	nd	nd	No	2.1	2.0
5	HGSC	PR	Yes	c.734G > A	WT	WT	WT	No	1.9	1.1
6	HGSC	CR	Yes	WT	c.415C > A	WT	*MUTYH* c.991G > T	No	1.5	1.5
7	UC	PR	Yes	c.245delC	c.5044G > T	c.8350C > T	WT	Yes	2.0	3.0
8	UC	DP	No	WT	c.3931_3934delAACA	WT	WT	Yes	2.3	1.9
9	HGSC	DP	Yes	nd	nd	nd	nd	Yes	2.6	1.1
10	HGSC	PR	Yes	c.536A > G c.4G > T	WT	WT	*POLE* c.4111C > T	No	1.5	1.2
11	HGSC	PR	Yes	c.920-2A > T	WT	WT	*APC* c.5758C > T c.6610C > T	Yes	2.0	1.5
12	HGSC	DP	No	c.718delA	WT	WT	*XRCC2* c.110C > A	No	3.5	3.2
13	HGSC	SD	No	nd	nd	nd	nd	No	1.7	1.8
14	HGSC	DP	Yes	nd	nd	nd	nd	No	2.4	2.0
15	HGE	DP	Yes	c.524G > A	c.5096G > A	WT	WT	No	2.2	1.1

^a^Target-next generation sequencing was performed in 11 of 15 cases. *HRD* homologous recombination deficiency, *CN* copy number, *HGSC* high grade serous carcinoma, *UC* undifferentiated carcinoma, *HGE* high grade endometrioid carcinoma, *CR* complete response, *PR* partial response, *SD* stable disease, *DP* disease progression, *nd* not determined, *WT* wild type alleles. Copy number gain was defined as CN > 2.0; copy number loss was defined as CN < 2.0. HRD was considered with CS score ≥ 42

**Table 3:** Homologous Recombination Deficiency (HRD) scores evaluated in 15 high grade ovarian cancer samples

ID	tAI	LOH	LST	CS
1	11	4	15	30
2	13	5	10	28
3	15	3	9	27
4	36	25	22	83
5	48	28	28	104
6^a^	39	15	30	84
7^a^	33	22	32	87
8^a^	12	5	13	30
9	35	14	28	77
10	40	19	26	85
11	15	0	28	43
12	15	6	9	30
13	31	9	24	64
14	8	4	5	17
15^a^	33	16	29	78
Median (P25–75)	31.0 (13–36)	9.0 (4–19)	24.0 (10–28)	64 (30–84)

*tAI* elomeric allellic imbalance score, *LOH* loss of heterozygosity score, *LST* large scale transition score, *CS* composite score, *P25–75* interquartile range. The cut-off values were the median 30 for tAI, > 10 for LOH, > 42 for CS and > 15 for near-diploid tumors or > 20 for near-tetraploid tumors for LST scores

^a^*BRCA1* mutated

The tNGS performed in 11 ovarian cancer revealed 18 somatic pathogenic variants including eight cases with *TP53* mutations, three with *BRCA1*, and one UC presented *BRCA1* and *BRCA2* mutations. Detailed information on *BRCA1* and *BRCA2* mutations are presented in Additional file 4. Pathogenic variants were also identified in *XRCC2*, *MUTYH*, *POLE* and *APC* genes (Table 2). Moreover, two *TP53* variants (c.536A > G and c.524G > A) identified in two tumors were covered by the OncoScan® FFPE platform, confirming the tNGS results. The mutational profiling of all cases of our study was obtained by tNGS performed in tumor samples and the allelic frequency detected for the genes tested is compatible with somatic mutations.

Three of four *BRCA1* mutated tumors showed high HRD scores pointing to homologous recombination deficiency. Two of these four *BRCA1* mutated tumors also presented gains of *CCNE1* (CN > 2.0). In addition, two cases with *XRCC2* mutation presented low HRD score and the highest value for *CCNE1* gains (CN = 4.0 and 3.5). Eight of 15 patients had *CCNE1* copy number gain and 11 of 15 patients presented *RB1* copy number loss (Table 2). Five of eight patients with *CCNE1* gains presented low HRD scores (Table 2).

### Overall response rate (ORR), progression free survival (PFS) and overall survival (OS)

Considering the entire cohort (*N* = 31), ORR was 51.6%, PFS was 7.6 months and OS was 13.4 months. Among the 15 patients molecularly evaluated (SNP array), two presented complete response, five showed partial response, two stable disease and six disease progression (ORR = 46.7%, PFS = 8.8 months and OS = 10.2 months) (detailed data are presented in Table 4).

**Table 4:** Review of the response to platinum retreatment in patients who were tested with tNGS (11 cases) and OncoScan® FFPE platform (15 cases)

ID	Pre-treatment CA125	Post-treatment CA125	CA125 Confirmation	Largest lesion pre -treatment	Largest lesion post-treatment	Treatment Response
1	102.9	32.2	–	SUV 4.54 in pelvis	No PET-CT contrast enhancement	CR
2	115	62	31	Pleural (72 mm)	Pleural (70 mm)	PR
3	40.9	20.1	17.1	Peritoneal (31 mm)	Peritonium (26 mm)	SD
4	637	1143	–	Liver (45 mm)	Liver (54 mm)	DP
5	92.3	79	24.2	Peritoneal (63 mm)	Peritonium (47 mm)	PR
6	35	18.6	0	SUV 11.0 in liver, 3.2 in adrenal, 8.0 pre-sacral	No PET-CT contrast enhancement	CR
7	1174	222,9	71.5	Pleural effusion	Pleural effusion resolution*	PR
8	258	331	–	SUV 3.0 in peritoneal lesion, 1.9 in epiplon, 3.0 in gut	SUV 11.6 in peritoneal lesion, 3.7 in epiplon, 9.5 in gut	DP
9	–	–	–	–	Bowel obstruction	DP
10	4430	1887	2180	Pelvic (101 mm)	Pelvic (90 mm)	PR
11	546	81.6	60	Pre-sacral (117 mm)	Pre-sacral (106 mm)	PR
12	2600	3004	–	Peritoneal thickening	New hepatic lesion (24 mm)	DP
13	32	14.2	17.6	Retroperitoneal lesion (40 mm)	Retroperitoneal lesion (40 mm)	SD
14	–	–	–	–	Bowel obstruction	DP
15	52.2	94.6	185	–	New brain lesion	DP

CA125 confirmation: a second CA125 measurement at least two weeks after the post treatment was performed aiming to confirm the treatment response; *PET-CT* FDG positron emission tomography – compute tomography, *SUV* standardized uptake value, *CR* complete response, *PR* partial response, *SD* stable disease, *DP* disease progression

*No surgical procedure was performed between the evaluations

**Fig. 1:**
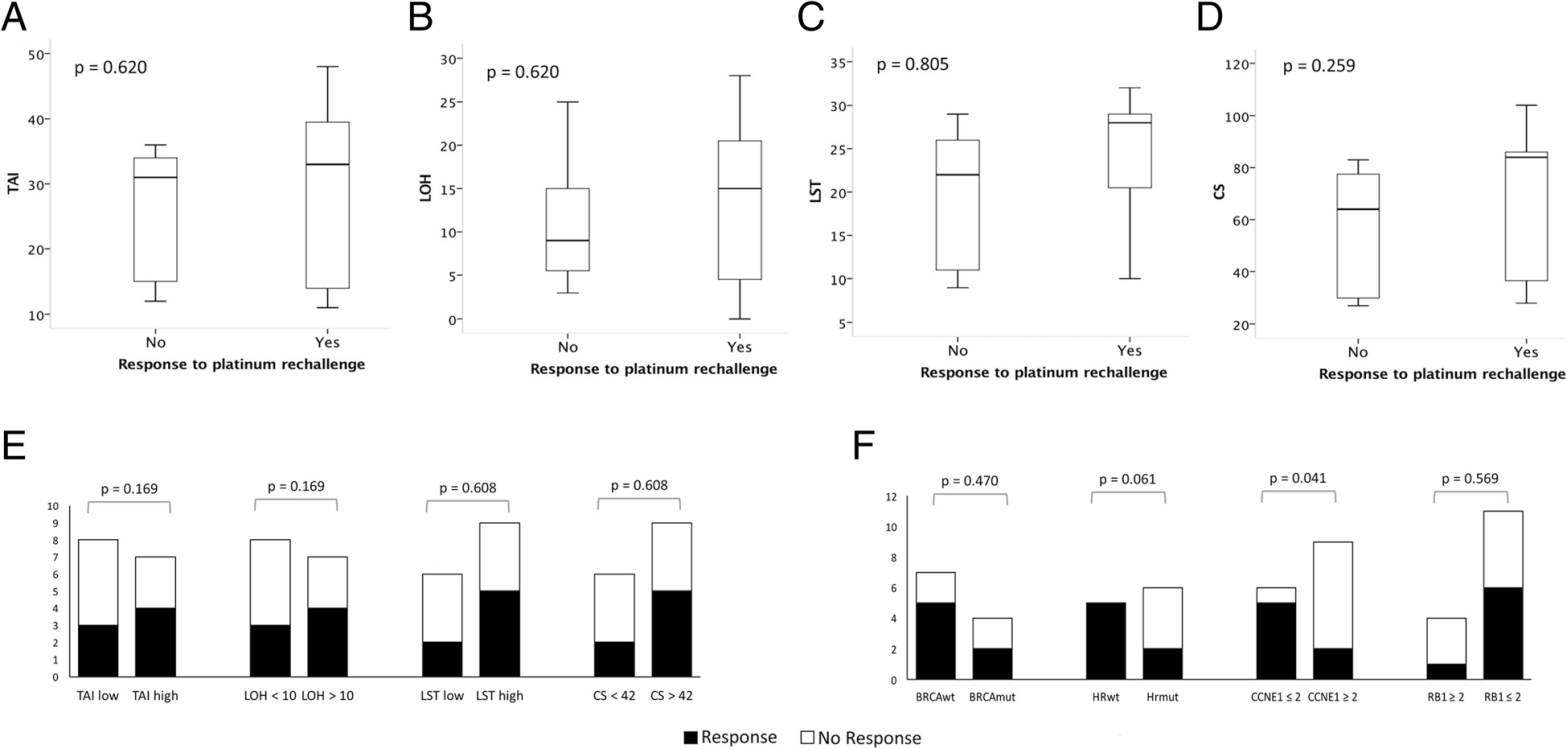
Homologous recombination deficiency scores according to the response to platinum rechallenge. **a** Telomeric allelic imbalance (tAI); **b** Loss of heterozygosity score (LOH); **c** Large scale transition score (LST); **d** Composite score (CS). Overall response rates according to molecular alterations. **e** Telomeric allelic imbalance score (tAI) Loss of heterozygosity score (LOH); Large scale transition score (LST) and Composite score (CS); **f** *BRCA* mutation; Homologous recombination (HR) gene mutation; CCNE1 copy number gain; *CCNE1* copy number gain and *RB1* copy number loss

The median values of the four HRD scores were higher in responders (LOH = 15, LST = 28, tAI = 33, CS = 84) compared with non-responders (LOH = 7.5, LST = 17.5, tAI = 23, CS = 47) (Fig. 1). Based on the cutoff values, patients with high LOH, LST, tAI and CS scores had better response rates, although these differences were not statistically significant (Fig. 1). Interestingly, two of four *BRCA1* mutated cases presented a response to platinum retreatment while in two patients with *XRCC2* mutation, one presented a stable disease and the other tumor progression (Fig. 1).

Ovarian cancer samples with no *CCNE1* CN gains were associated with higher response rate compared with cases with *CCNE1* CN gains (ORR = 83.3 vs 22.2%; *p* = 0.041). Although not statistically significant, patients with *RB1* CN loss had higher response rates compared with patients without *RB1* CN loss (ORR 54.5% versus 25.0%). Two patients with *BRCA1* mutation showing no response to platinum retreatment also presented *CCNE1* CN gain (Fig. 1)*.* CNA of both, *RB1* (loss) and *CCNE1* (gains) were observed in 6 cases.

**Fig. 2:**
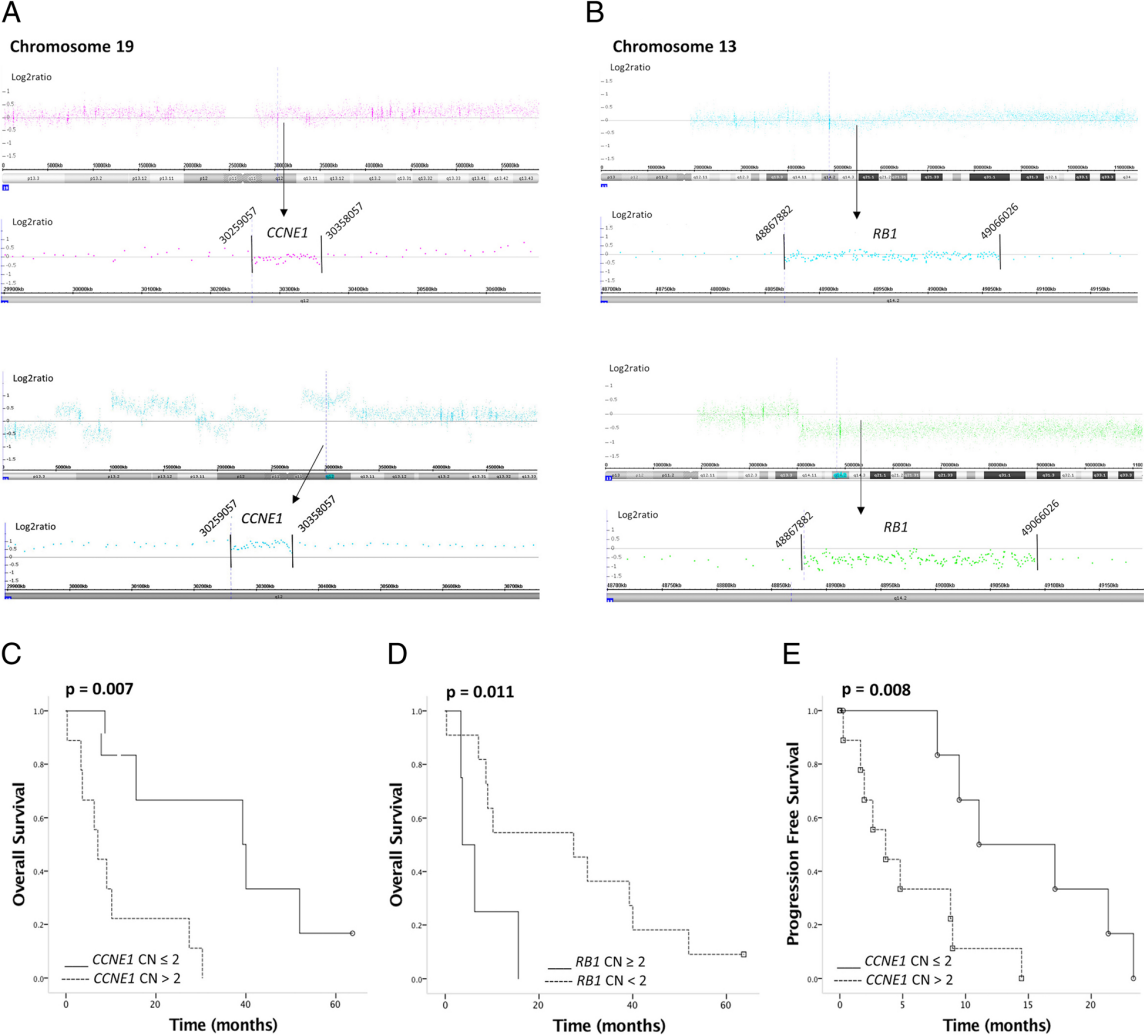
Representative examples of *CCNE1* copy number status. **a** *CCNE1* copy number normal was observed in 7 cases. **b** *CCNE1* copy number gain was detected t in 8 cases. **c** Overall survival according to *CCNE1* copy number alteration and **d** *RB1* copy number alteration. **e** PFS according to *CCNE1* copy number alteration

No significant differences were observed in the PFS and OS according to the homologous recombination deficiency scores (Additional files 5 and 6). However, *RB* and *CCNE1* CNAs were significantly associated with these parameters. Patients with *CCNE1* gains presented worse PFS compared with patients without *CCNE1* gains (PFS = 7.1 months vs 39.3 months; *p* = 0.007) (Fig. 2). Patients with *RB1* losses had better PFS compared with cases with no *RB1* losses (PFS = 9.0 months vs 2.6 months; *p* = 0.093) (Additional file 6).

Cases with *CCNE1* CN gains presented worse overall survival compared to those with no *CCNE1* CN gains (OS = 39.3 months vs 7.1 months; p = 0.007). In contrast, better overall survival was found in cases with *RB1* CN loss compared with those with no *RB1* CN loss (OS = 27.4 months versus 3.6 months; *p* = 0.025) (Fig. 2).

## Discussion

In this study, we evaluated markers of response to platinum retreatment in a selected group of patients with heavily pretreated platinum resistant ovarian cancer. The rationale was to identify patients that are still sensitive even after a platinum free interval shorter than 6 months and numerous previous treatment lines.

Nine of 15 cases (60%) of our cohort presented HR deficiency, which is in accordance with the 50% described in the TCGA dataset [3]. Patients with CS score higher than 42 had an ORR of 55.6% versus 33.3% observed in those with lower scores. In addition, the HRD median values of each score were higher among responders than in non-responders. However, the differences were not statistically significant probably due to the small number of cases evaluated.

Two scores based on the patterns of CNA and LOH were used in the phase III clinical trials of PARP inhibitors showing their ability to identify patients with *BRCA* mutation [7, 8, 13]. These studies also described a second group of patients negative for *BRCA* mutations but with high sensitivity to PARP inhibitors. Our findings give additional evidence that the scores are able to identify patients with high sensitivity to platinum agents. The absence of statistical significance in our study may be due to the small number of cases or the accuracy of these scores. Interestingly, the *BRCA* non-mutated cases and those with low HRD (based on the scores) benefited from treatment with PARP inhibitors [7, 8, 13]. The definition of all scores, except tAI [14], were based on the *BRCA* mutation as a gold standard to define HRD [15–17]. Therefore, the CNA and LOH pattern calculated by the scores are similar to the ones promoted by *BRCA* mutations, which may be true for most but not necessarily all causes of the HRD.

Cyclin E1 overexpression promotes cell cycle progression abrogating DNA repair during the G1 phase. In addition, *CCNE1* amplification has been associated with an apparent synthetic lethality in cases with HR deficiency [28]. Nine of our 15 tumors (60%) showed *CCNE1* copy number gains while data from TCGA (509 high grade serous ovarian carcinomas) presented *CCNE1* gains in 32.4% or focal amplification in 20.8% [3]. We observed that cases showing *CCNE1* gains had lower ORR, and shorter PFS and OS compared with those not presenting gains. Previous studies in ovarian cancer patients described increased expression levels of cyclin E1 and an association with worse survival [29]. At the genomic level, two studies described high frequency of *CCNE1* gains in patients with primary platinum resistant disease [19, 30]. The worse survival observed in the TCGA cohort for patients with *CCNE1* amplification was attributed to its negative association with *BRCA* mutations [3]. In our study, two of four patients harboring *BRCA* mutations also presented *CCNE1* gains. This finding suggests that the correlation between *CCNE1* gain and outcome is not exclusively due to its negative association with *BRCA* mutations. Ten of our 15 patients had primary platinum resistant disease and 7 of 9 patients with *CCNE1* gains presented primary resistant disease. This finding supports the association of *CCNE1* aberrations and resistance to platinum therapy and may explain the higher than expected frequency of *CCNE1* gains in our study. Previous studies showed *CCNE1* gains and *BRCA* mutation or homologous recombination deficiency as mutually exclusive [29]. However, the authors showed that complete mutually exclusive alterations were not observed between low levels of *CCNE1* gains and *BRCA* mutations. Our two patients with co-occurrence of *BRCA* mutation and *CCNE1* gain presented low *CCNE1* copy number gains (2.2 and 2.3).

The RB1 protein is involved in the S-phase checkpoint to repair DNA breaks and in the regulation of DNA replication. The loss of the tumor suppressor *RB1* leads to cell cycle progression and replication fork progression leading to replication stress and DNA damage, which could be repaired by HR machinery [31]. We found an association of *RB1* loss with PFS and OS. To our knowledge, two previous studies addressed the impact of *RB1* loss in ovarian carcinoma. In 2013, Milea et al. showed loss of RB1 protein expression associated with longer OS [20]. Recently, Garsed et al. reported the association of *RB1* loss and HR gene mutations with extremely long PFS and OS [21]. Taken together, these findings suggest that *RB1* loss is a biomarker with the potential to identify sensitive patients to platinum treatment. In addition, this alteration could be used in the clinical practice and potentially select *BRCA* mutated patients with higher chance to be PARP inhibitors responsive.

Four of 15 tumors (26%) presented *BRCA* mutations, an expected frequency in high grade serous carcinomas [32]. No differences in the response of the treatment were found between mutated and non-mutated tumors. Although, *BRCA* mutations are well known markers of response to platinum and PARP inhibitors therapy, other studies also failed to show higher frequency of *BRCA* mutations in long term responders [21]. In addition to *BRCA*, two non-responder patients presented tumors with low HR deficiency scores and *XRCC2* mutations. *XRCC2* is involved in the repair of DNA double-strand breaks by HR pathway. This finding highlights the value of an investigation using a panel of genes in ovarian cancer. Furthermore, conclusions regarding HR deficiency based solely on the presence of certain mutations may not be precise, as was found in the ARIEL2 trial [13].

Our study has several limitations, mostly due to the small sample size and its retrospective nature. For example, we found the ORR higher than expected for platinum resistant patients. High ORR may be due to patient over-selection including those who have received several previous treatment lines. Despite the limitation of the ORR evaluation, *CCNE1* gain and *RB1* loss were both associated to OS, which is an objective endpoint even in retrospective studies. No association between HR deficiency scores and response to therapy was found. The small number of patients limits conclusions regarding the low accuracy of these scores, even if previous literature data also pointed out to the limitation of the scores. However, the strength of our study was the evaluation of well selected individuals for whom it would be expected a low response rate with platinum retreatment. Unexpectedly, a high response rate to platinum retreatment was found suggesting that this selected cohort might be enriched for extremely sensitive tumors. HR deficiency scores were not able to show a strong association with therapy response. Interestingly, *CCNE1* copy number gain was a negative prediction marker of platinum sensitivity, and *RB1* copy number loss identified patients with sensitive disease.

In this study we explored the mutational profile and HR deficiency score in ovarian cancer patients to better understand the platinum resistant recurrence as defined by the platinum free interval. We demonstrated that HR deficiency scores, CCNE1 gains and RB1 losses could be used to distinguish patients who are still sensitive to platinum retreatment from those resistant to platinum therapy. Considering similar mechanisms of sensitivity to platinum salts and PARP inhibitors, these markers could be useful to better select the patients for PARP inhibitors therapy in the platinum resistant relapse.

## Conclusions

The prediction of response to platinum retreatment goes beyond HR deficiency. *CCNE1* copy number gains revealed a different subtype of ovarian carcinoma and could be used as a negative selection marker for platinum therapy and PARP inhibitors. Furthermore, *RB1* losses identified patients with higher chance to be responsive to the treatment. Theses markers add information to precision therapy in the context of the cost-effectivity for PARP inhibitors therapy.

## Additional files

Additional file 1: Flowchart representative of the inclusion criteria adopted in the study. (JPG 245 kb)

Additional file 2: Summary of the bioinformatic pipeline used to classify the variants detected by tNGS. (JPG 509 kb)

Additional file 3: Spearman correlation of four different homologous recombination deficiency scores. TAI, Telomeric allelic imbalance; LOH, Loss of heterozygosity score; LST, Large scale transition score; CS, Composite score. (JPG 353 kb)

Additional file 4: *BRCA1* and *BRCA2* variants categorized according to ACMG (*American College of Medical Genetics and Genomics*). (DOCX 14 kb)

Additional file 5: Progression free survival according to the molecular alterations. A. Telomeric allelic imbalance (tAI); B. Loss of heterozygosity score (LOH); C. Large scale transition score (LST); D. Composite score (CS); F. RB1 copy number gain. (JPG 170 kb)

Additional file 6: Overall survival according to the genomic imbalances. A. Telomeric allelic imbalance (TAI); B. Loss of heterozygosity score (LOH); C. Large scale transition score (LST); D. Composite score (CS). (JPG 374 kb)

## Abbreviations

CNA: Copy number alterations
CS: Composite score
FFPE: Formalin fixed paraffin embedded
GCIG: Gynecological Cancer Intergroup
HR: Homologous recombination
HRD: Homologous recombination deficiency
LOH: Loss of heterozigosity
LST: Large scale transition
NGS: Next generation sequencing
OS: Ooverall survival
PARP: Poly(ADP-ribose) polymerase
PFI: Platinum-free interval
PFS: Progression free survival
RECIST: Response evaluation criteria in solid tumors
SNP: Single nucleotide polymorphism
tAI: Telomeric allelic imbalance
