# Supplementary figures and images for: Genomic profiling in ovarian cancer retreated with platinum based chemotherapy presented homologous recombination deficiency and copy number imbalances of CCNE1 and RB1 genes
Source: BMC Cancer. 2019 May 6;19:422. doi: 10.1186/s12885-019-5622-4 (PMC6503431; doi:10.1186/s12885-019-5622-4)

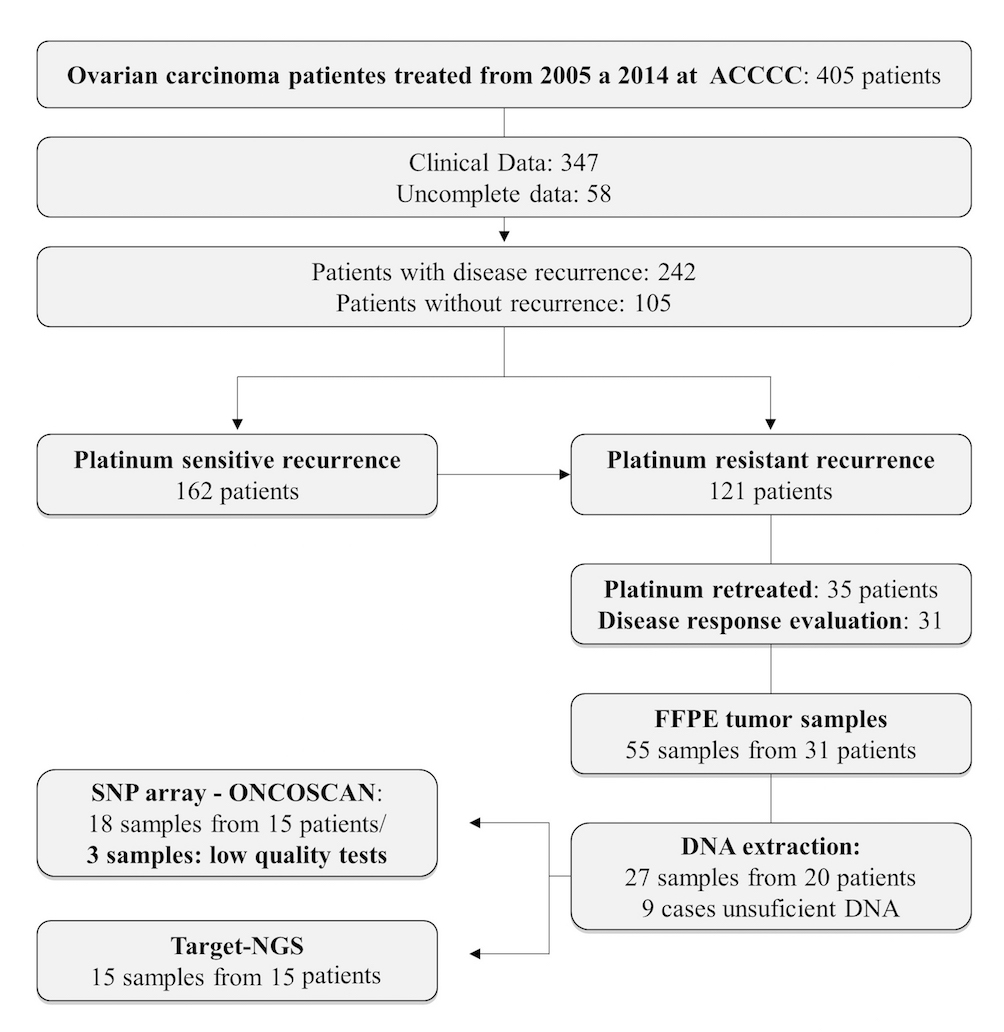

Supplement: Supplementary file 1 — Flowchart representative of the inclusion criteria adopted in the study. (JPG 245 kb) [file 12885_2019_5622_MOESM1_ESM.jpg]

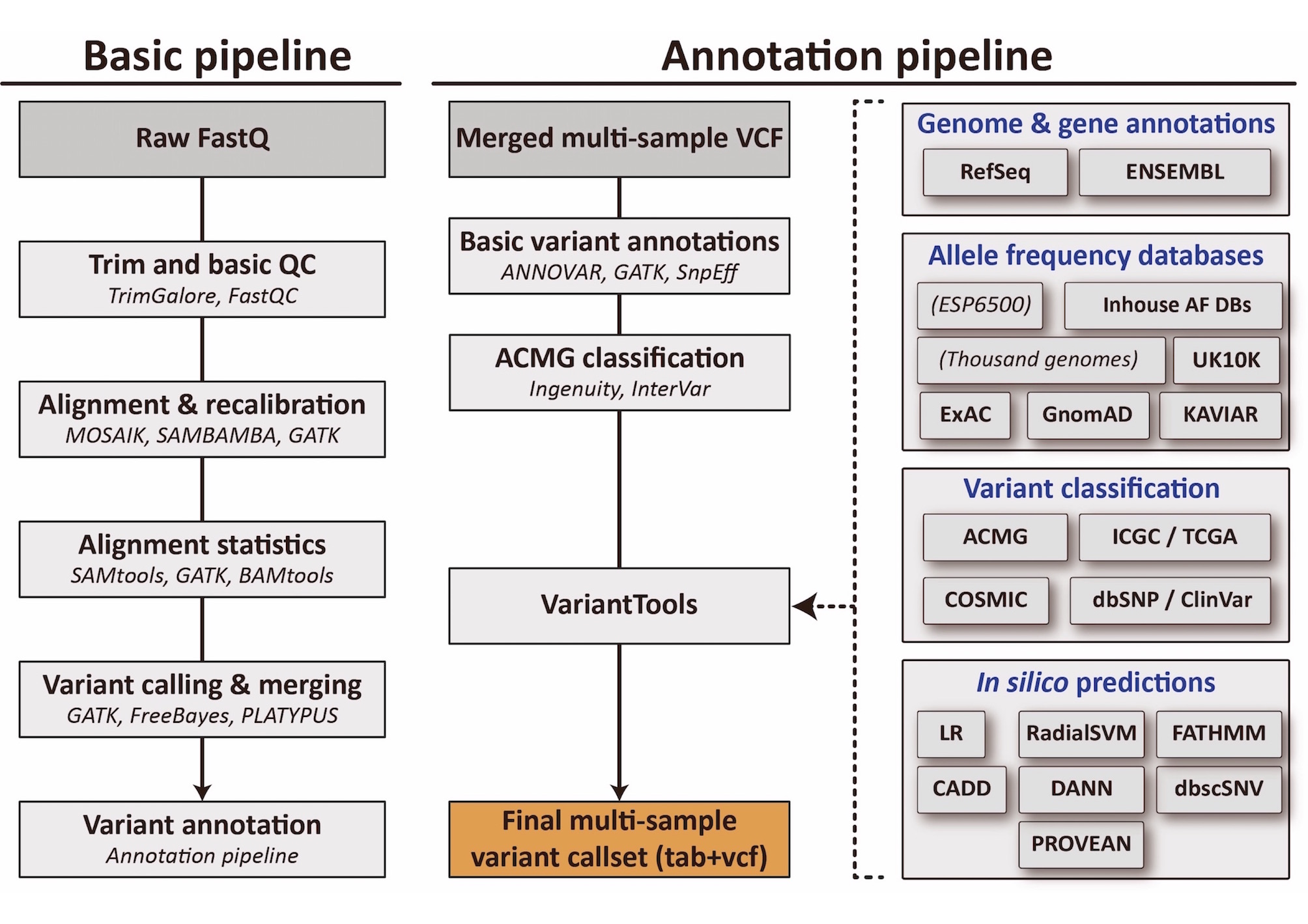

Supplement: Supplementary file 2 — Summary of the bioinformatic pipeline used to classify the variants detected by tNGS. (JPG 509 kb) [file 12885_2019_5622_MOESM2_ESM.jpg]

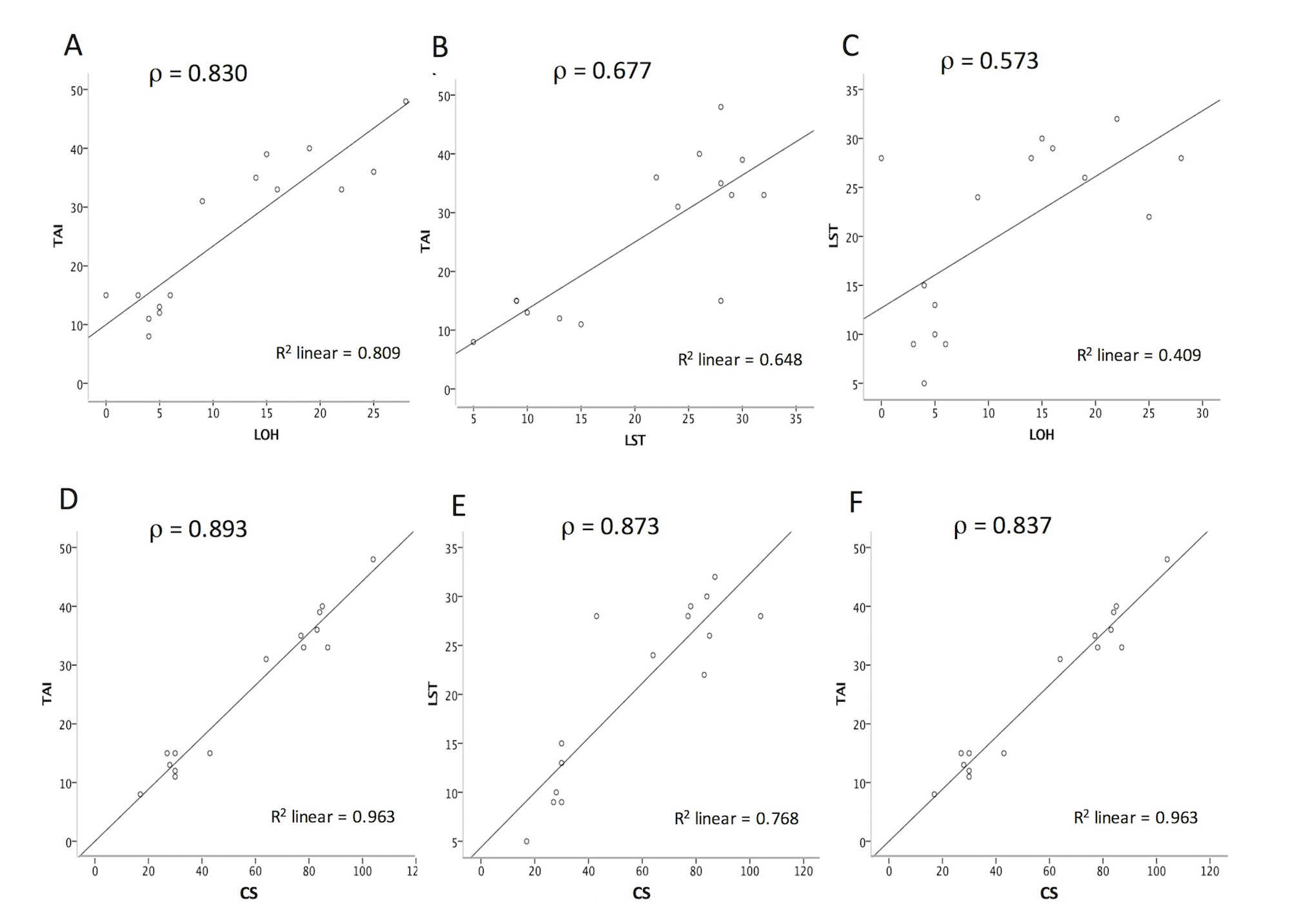

Supplement: Supplementary file 3 — Spearman correlation of four different homologous recombination deficiency scores. TAI, Telomeric allelic imbalance; LOH, Loss of heterozygosity score; LST, Large scale transition score; CS, Composite score. (JPG 353 kb) [file 12885_2019_5622_MOESM3_ESM.jpg]

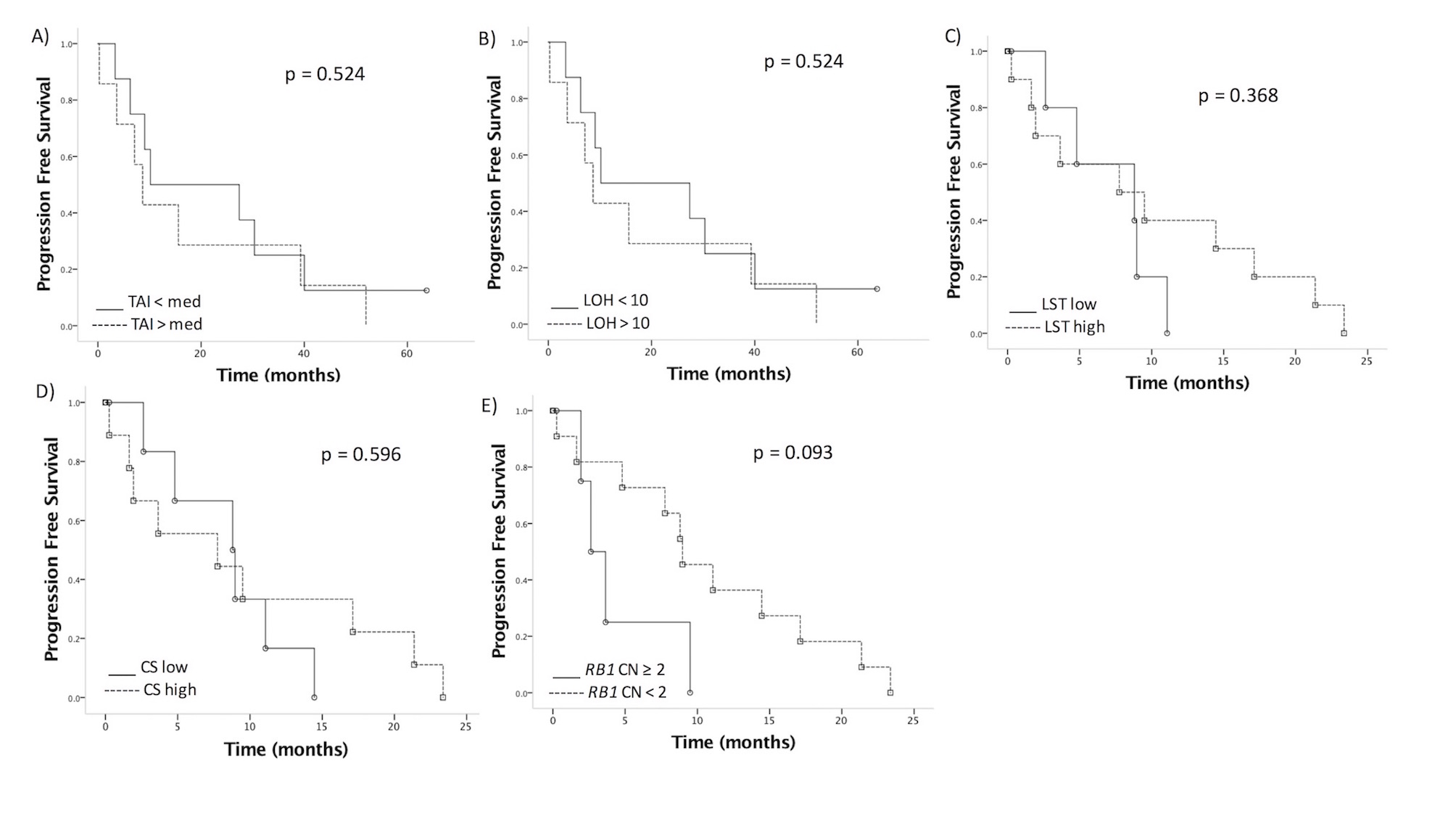

Supplement: Supplementary file 5 — Progression free survival according to the molecular alterations. A. Telomeric allelic imbalance (tAI); B. Loss of heterozygosity score (LOH); C. Large scale transition score (LST); D. Composite score (CS); F. RB1 copy number gain. (JPG 170 kb) [file 12885_2019_5622_MOESM5_ESM.jpg]

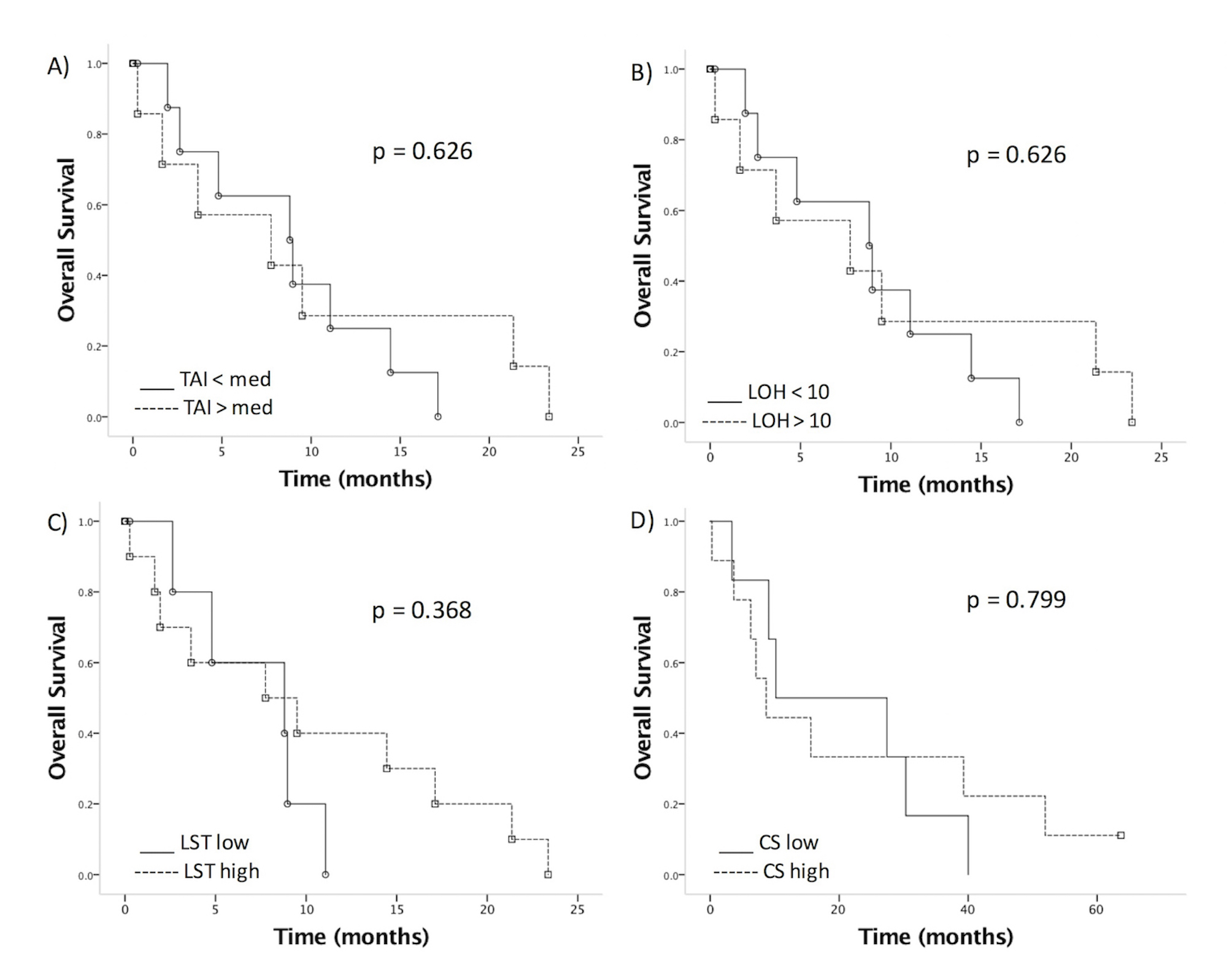

Supplement: Supplementary file 6 — Overall survival according to the genomic imbalances. A. Telomeric allelic imbalance (TAI); B. Loss of heterozygosity score (LOH); C. Large scale transition score (LST); D. Composite score (CS). (JPG 374 kb) [file 12885_2019_5622_MOESM6_ESM.jpg]
