# Supplementary material for: Genomic profiling in ovarian cancer retreated with platinum based chemotherapy presented homologous recombination deficiency and copy number imbalances of CCNE1 and RB1 genes
Source: BMC Cancer. 2019 May 6;19:422. doi: 10.1186/s12885-019-5622-4 (PMC6503431; doi:10.1186/s12885-019-5622-4)
Supplement: Supplementary file 4 — BRCA1 and BRCA2 variants categorized according to ACMG (American College of Medical Genetics and Genomics). (DOCX 14 kb) [file 12885_2019_5622_MOESM4_ESM.docx]

| **Gene Names** | **dbSNP**  **Ref/Alt** | **Identifier** | **ACMG Classification** | **Sequence Ontology (Combined)** | **Effect (Combined)** | **HGVS c. (Clinically Relevant)** | **HGVS p. (Clinically Relevant)** |
| --- | --- | --- | --- | --- | --- | --- | --- |
| *BRCA2* | C/T | [rs80359075](http://www.ncbi.nlm.nih.gov/projects/SNP/snp_ref.cgi?rs=rs80359075) | VUS/Weak Pathogenic | missense_variant | Missense | NM_000059.3: c.8350C>T | NP_000050.2:p.Arg2784Trp |
| *BRCA1* | C/A/G/T | [rs41293459](http://www.ncbi.nlm.nih.gov/projects/SNP/snp_ref.cgi?rs=rs41293459) | VUS/Weak Pathogenic | missense_variant | Missense | NM_007294.3: c.5096G>A | NP_009225.1:p.Arg1699Gln |
| *BRCA1* | . | . | Likely Pathogenic | stop_gained | Loss of Function | NM_007294.3: c.5044G>T | NP_009225.1:p.Glu1682Ter |
| *BRCA1* | TGTT/- | [rs80357864](http://www.ncbi.nlm.nih.gov/projects/SNP/snp_ref.cgi?rs=rs80357864) | VUS/Weak Pathogenic | frameshift_variant | Loss of Function | NM_007294.3: c.3931_3934delAACA | NP_009225.1:p.Asn1311Profs*6 |
| *BRCA1* | . | . | VUS/Conflicting | missense_variant | Missense | NM_007294.3: c.415C>A | NP_009225.1:p.Gln139Lys |

**Additional file 4.** *BRCA1* and *BRCA2* variants categorized according to ACMG (*American College of Medical Genetics and Genomics*)
